# Predictive Value of the Soluble fms-Like Tyrosine Kinase-1/Placental Growth Factor Ratio in Preeclampsia and Placenta Accreta Spectrum: A Prospective Observational Study

**DOI:** 10.7759/cureus.113695

**Published:** 2026-07-30

**Authors:** Ashima Taneja, Guntas Kaur, Surbhi Handa, Sukriti Gupta

**Affiliations:** 1 Department of Obstetrics and Gynaecology, Dayanand Medical College and Hospital, Ludhiana, IND

**Keywords:** angiogenic biomarkers, placenta accreta spectrum, placental growth factor, preeclampsia, soluble fms-like tyrosine kinase-1

## Abstract

Background: Certain placentation disorders, such as preeclampsia and placenta accreta spectrum (PAS), are prevalent causes of maternal and fetal morbidity. Angiogenic biomarkers, including soluble fms-like tyrosine kinase-1 (sFlt-1), placental growth factor (PlGF), and the sFlt-1/PlGF ratio, are increasingly used in the evaluation of preeclampsia; however, their relevance in PAS remains unclear.

Objective: To evaluate the diagnostic performance of sFlt-1, PlGF, and the sFlt-1/PlGF ratio for preeclampsia and explore their potential diagnostic utility in PAS.

Methodology: This prospective observational study was conducted at a tertiary care hospital in North India from June 2024 to June 2025. A total of 200 antenatal women at 20-37 weeks of gestation were recruited and divided into three groups: preeclampsia, PAS, and a comparison group without preeclampsia or PAS. Serum levels of sFlt-1 and PlGF were determined by electrochemiluminescence immunoassay (ECL), and the ratio sFlt-1/PlGF was calculated. The optimum cutoff values were determined by the receiver operating characteristic curve, and the diagnostic performance was evaluated.

Results: Of the 200 participants, 57 (28.5%) had preeclampsia, 16 (8.0%) had PAS, and 127 (63.5%) had neither condition. At a cutoff of 77.65, the sFlt-1/PlGF ratio performed the best, with an area under the curve of 0.902 for preeclampsia, an accuracy of 88.0%, a positive predictive value (PPV) of 80.7%, a negative predictive value (NPV) of 91.3%, and a sensitivity of 80.7%. PAS had a high NPV of 97.1%, a low PPV of 18.7%, a high sensitivity of 87.5%, a low specificity of 52.0%, an accuracy of 55.9%, and an area under the curve (AUC) of 0.678.

Conclusions: The sFlt-1/PlGF ratio was an excellent discriminator of preeclampsia and could be used to help identify and stratify at-risk individuals. It is not very specific and has a low PPV in PAS, meaning that it should not be used as a surrogate for imaging-based diagnosis and clinical risk assessment in PAS.

## Introduction

Globally, preeclampsia is a leading cause of maternal and perinatal morbidity and mortality. It complicates 2%-8% of pregnancies in developed countries and is reported to be more common in developing countries, reaching as high as 16.7% [1,2]. Preeclampsia is a major cause of poor obstetric-gynecologic outcomes worldwide, causing over 70,000 pregnant women and over 500,000 fetuses to die each year [3]. Clinical significance is that, when the process of disease progresses, it could result in a range of complications such as placental abruption, acute kidney injury, cerebral hemorrhage, hepatic dysfunction, pulmonary edema, disseminated intravascular coagulation, and eclampsia [4]. Fetal and neonatal complications are fetal growth restriction, oligohydramnios, preterm delivery, stillbirth, respiratory distress syndrome, necrotizing enterocolitis, and intraventricular hemorrhage.

Preeclampsia is closely associated with abnormal placentation, impaired spiral artery remodeling, endothelial dysfunction, and angiogenic imbalance. Increased markers of placental dysfunction, such as soluble fms-like tyrosine kinase-1 (sFlt-1), and decreased markers, such as placental growth factor (PlGF), are among the most studied circulating biomarkers. The sFlt-1/PlGF ratio represents the balance between antiangiogenic and proangiogenic activity, and this ratio has been included in clinical risk assessment of suspected preeclampsia. It can be used in conjunction with earlier diagnosis, better short-term risk stratification, and decision-making for surveillance, and may help in the timing of delivery.

Placenta accreta spectrum (PAS) is another abnormal placentation disorder, characterized by accreta, increta, and percreta (based on the depth of the placentation or uterine wall invasion) [5]. Its incidence is around 0.17% (1-2 cases per 1,000 deliveries) [6]. This is accompanied by significant maternal morbidity, especially due to large obstetric bleeding. The average blood loss can be greater than 3,000 mL, over 90% of affected women may need a blood transfusion, and a large percentage of women will have a peripartum hysterectomy [1]. Other maternal complications include bladder injury, sepsis, intensive care admission, and mortality, while fetal risks are primarily preterm birth and fetal growth restriction [7].

Enhancing the clinical risk assessment and imaging are currently the only methods used to predict PAS antenatally. The use of transabdominal or transvaginal ultrasonography with color Doppler may show placental lacunae, loss of the retroplacental clear zone, myometrial thinning, and abnormal uteroplacental vascularity [8]. Magnetic resonance imaging can provide additional anatomical information, especially when posterior placentation is suspected, extrauterine invasion is suspected, or the ultrasonographic findings are inconclusive. Previous cesarean section, placenta previa, previous uterine surgery, and maternal age continue to be the most important clinical risk factors for PAS and are used for risk stratification [9,10]. The use of angiogenic biomarkers has been well established in preeclampsia but is not established in PAS. PAS is also characterized by abnormal trophoblastic invasion and placental vascular remodeling; therefore, some biomarkers, including sFlt-1 and PlGF, could be of potential interest as additional markers, but their diagnostic value has not been sufficiently established [11,12].

Preeclampsia and PAS are two types of placentation disorder, but they are clinically important. Although both are conditions that need to be recognized in the antepartum period, aiming to minimize maternal and fetal complications, they have different pathophysiological characteristics, clinical manifestations, and existing diagnostic methods. The assessment of these angiogenic biomarkers in these two conditions could help determine whether these factors could be used for more detailed disease-specific diagnostic utility than that provided by current clinical and imaging parameters.

Objectives of the study

This study aimed to determine cohort-specific optimal cutoff values for sFlt-1, PlGF, and the sFlt-1/PlGF ratio and assess their diagnostic performance for preeclampsia using sensitivity, specificity, positive predictive value (PPV), negative predictive value (NPV), area under the receiver operating characteristic curve (AUC), and diagnostic accuracy (DA). A secondary, exploratory objective was to assess the diagnostic performance of these biomarkers for PAS, with the PAS findings interpreted as hypothesis-generating because of the small sample size and group imbalance. The additional aim was to evaluate the associations between these angiogenic markers and selected maternal and fetal complications.

This paper was previously posted to the Authorea preprint server on July 16, 2025, at https://www.authorea.com/doi/full/10.22541/au.175267084.48078768/v1 [13].

## Materials and methods

Study design and setting

This prospective observational study was carried out from June 2024 to June 2025 in the Department of Obstetrics and Gynaecology at a tertiary care hospital in North India. Pregnant women presenting between 20 and 37 weeks of gestation were assessed for eligibility. A total of 200 participants were enrolled after providing written informed consent. Women who had fetal congenital anomalies or intrauterine fetal demise at enrollment were excluded.

Study groups

Participants were classified into three mutually exclusive groups according to their final clinical diagnosis. Women with preeclampsia were included in Group A, women with PAS were included in Group B, and pregnant women without preeclampsia or PAS were included in Group C as the comparison group. Preeclampsia was diagnosed according to institutional clinical protocols, using maternal clinical status, blood pressure measurements, assessment of proteinuria, relevant laboratory evaluations, and specialist review. PAS was diagnosed using maternal risk factors, obstetric imaging, and specialist assessment, with operative or pathological findings considered when available.

Clinical assessment and follow-up

On enrollment, all participants underwent a detailed clinical assessment, including demographic evaluation, obstetric history, assessment of relevant maternal risk factors, general physical examination, and obstetric examination. Routine antenatal investigations were performed according to institutional protocol. The gestational age at biomarker testing was documented. Biomarker testing was performed at a single time point after enrollment. All participants were followed through delivery, and the interval between biomarker testing and delivery was recorded.

Biomarker assessment

Blood samples were collected for the measurement of serum sFlt-1 and PlGF concentrations. Elecsys® sFlt-1 and Elecsys® PlGF reagent kits, both based on electrochemiluminescence immunoassay technology, were used for biomarker analysis according to the manufacturer’s instructions. The results were reported in picograms per milliliter (pg/mL). The minimum detectable concentration was 10 pg/mL for sFlt-1 and 3 pg/mL for PlGF, as indicated by the manufacturer. The sFlt-1/PlGF ratio was calculated by dividing the measured sFlt-1 concentration by the measured PlGF concentration for each participant. A ratio <38 was considered within the normal range, and a ratio >85 was considered suggestive of preeclampsia. These reference thresholds were distinct from the cohort-specific thresholds subsequently derived through receiver operating characteristic curve analysis.

Outcome measures

Maternal and fetal outcomes were recorded during follow-up and after delivery. Fetal outcomes included fetal growth restriction, oligohydramnios, intrauterine demise, neonatal death, neonatal intensive care unit admission, and prolonged neonatal hospitalization. The gestational age at biomarker testing and the interval between biomarker testing and delivery were also analyzed.

Statistical analysis

Descriptive statistics included the mean and standard deviation for continuous variables and frequency and percentage for categorical variables. Continuous variables were assessed for distribution using the Kolmogorov-Smirnov test. Since the quantitative data did not satisfy the assumption of normality, intergroup comparisons of continuous variables were performed using the Kruskal-Wallis test. Categorical variables were compared using the chi-square test or Fisher’s exact test, as appropriate. Test statistic values were reported with corresponding *P*-values where applicable. Receiver operating characteristic curve analysis was used to derive cohort-specific optimal thresholds for sFlt-1, PlGF, and the sFlt-1/PlGF ratio for preeclampsia and PAS. Sensitivity, specificity, PPV, NPV, area under the curve (AUC) with standard error, and diagnostic accuracy were used to evaluate diagnostic performance. All analyses were based on the final diagnostic group assigned to each participant. The derived thresholds were specific to the present cohort and were not externally validated. No multivariable adjustment was performed for potential confounders, including gestational age, maternal age, placenta previa, prior cesarean delivery, disease severity, or other PAS risk factors; this limitation was considered when interpreting the findings. A *P*-value <0.05 was considered statistically significant. All analyses were performed using SPSS version 26.0 for Microsoft Windows (IBM Corp., Armonk, NY).

Ethical statement

The study was approved by the Institutional Research and Ethics Committee of Dayanand Medical College and Hospital, Ludhiana, June 12, 2023, vide letter no. DMCH/P/2023/556, June 21, 2023, with reference number DMCH/4/42-2022 (4505). Written informed consent was obtained from all participants before enrollment. All procedures were conducted in accordance with the Declaration of Helsinki.

## Results

Study population

The study involved a total of 200 pregnant women. Of these, 57 (28.5%) women had preeclampsia and were classified as Group A, 16 (8.0%) women had PAS and were classified as Group B, and 127 (63.5%) women had neither preeclampsia nor PAS and were classified as Group C.

Baseline characteristics

The mean maternal age, body mass index (BMI), gestational diabetes mellitus status, and period of gestation at biomarker testing were comparable among the three groups. No statistically significant intergroup differences were observed for mean age (*H* = 1.572, *P* = 0.704), BMI (*H* = 2.694, *P* = 0.070), gestational diabetes mellitus status (Fisher’s exact test, *P* = 0.219), or period of gestation at testing (*H* = 0.444, *P* = 0.642). Gravidity differed significantly across groups, with primigravida status more frequent in Group A and multigravida status more frequent in Group B (χ² = 24.371, *P* = 0.001). Socioeconomic status also differed significantly among the groups (χ² = 30.770, *P* = 0.001).

Fetal outcomes

Fetal growth restriction, oligohydramnios, intrauterine demise, and NICU admission differed significantly among the groups. Fetal growth restriction was most frequent in Group A and absent in Group B (Fisher's exact test, *P* = 0.020). Oligohydramnios was also more frequent in Group A and absent in Group B (Fisher's exact test, *P* = 0.025). Intrauterine demise differed significantly among the groups, occurring in six (10.5%) women in Group A, one (6.3%) woman in Group B, and 0 (0%) women in Group C (Fisher’s exact test, *P* = 0.001). NICU admission occurred in 27 (47.4%) neonates in Group A, 0 (0%) in Group B, and 33 (26.0%) in Group C (Fisher’s exact test, *P* = 0.020).

Neonatal death and prolonged hospital stay did not differ significantly among the groups (Fisher's exact test, *P* = 0.509 and *P* = 0.429, respectively). Baseline characteristics and fetal outcomes of the three study groups are shown in Table 1.

**Table 1:** Demographic characteristics, maternal characteristics, and fetal outcomes. Data are presented as mean ± standard deviation for continuous variables and as *n* (%) for categorical variables. Continuous variables were compared using the Kruskal-Wallis test. Categorical variables were compared using the chi-square test or Fisher’s exact test, as appropriate. Fisher's exact test statistic values are shown as obtained from the statistical output. PAS, placenta accreta spectrum; NICU, neonatal intensive care unit; BMI, body mass index; GDM, gestational diabetes mellitus; FGR, fetal growth restriction; SD, standard deviation; *H*, Kruskal-Wallis test statistic; χ², chi-square test statistic

Parameters	Group A (*n* = 57), 28.5%	Group B (*n* = 16), 8.0%	Group C (*n* = 127), 63.5%	Statistical parameter	*P*-value
Maternal characteristics					
Mean age (years)	30.67 ± 4.74	29.94 ± 3.92	30.12 ± 4.31	*H* = 1.572	0.704
Gravida	Primigravida: 40 (70.2%)	Multigravida: 1 (93.8%)	Multigravida: 53 (41.7%)	χ² = 24.371	0.001
Socioeconomic status	Upper class: 25 (43.9%)	Upper class: 8 (50.0%)	Lower class: 28 (27.6%)	χ² = 30.770	0.001
BMI (kg/m²)	30.93 ± 3.77	30.34 ± 1.86	29.52 ± 4.06	*H* = 2.694	0.070
Gestational diabetes	5 (8.8%)	0 (0%)	9 (7.1%)	Fisher’s exact test = 3.034	0.219
Fetal complications					
Fetal growth restriction	21 (36.8%)	0 (0%)	10 (7.9%)	Fisher’s exact test = 7.791	0.020
Oligohydramnios	11 (19.3%)	0 (0%)	8 (6.3%)	Fisher’s exact test = 11.111	0.025
Intrauterine demise	6 (10.5%)	1 (6.3%)	0 (0%)	Fisher’s exact test = 13.296	0.001
Neonatal death	4 (7.0%)	0 (0%)	6 (4.7%)	Fisher’s exact test = 1.351	0.509
Prolonged hospital stay	4 (7.0%)	0 (0%)	5 (3.9%)	Fisher’s exact test = 1.688	0.429
NICU admission	27 (47.4%)	0 (0%)	33 (26.0%)	Fisher’s exact test = 21.166	0.020
Period of gestation at testing (weeks)	31.91 ± 3.63	32.94 ± 3.02	32.13 ± 4.10	*H* = 0.444	0.642

Diagnostic performance for preeclampsia

The following sensitivity, specificity, PPV, NPV, and AUC were obtained for the threshold value of 5,212.00 pg/mL: 80.7%, 80.31%, 64.79%, 90.27%, and 0.862. PlGF AUC was 0.852, with a PPV of 68.75%, an NPV of 89.17%, a sensitivity of 77.19%, and a specificity of 84.25%. The best indicator of preeclampsia was the sFlt-1/PlGF ratio. It had a sensitivity of 80.7%, specificity of 91.34%, PPV of 80.70%, NPV of 91.34%, DA of 88.0%, and an area under the curve of 0.902 at a cutoff of 77.65.

Diagnostic performance for PAS

In PAS, sFlt-1 demonstrated poor diagnostic performance, with a sensitivity of 6.25%, specificity of 59.84%, PPV of 1.92%, NPV of 83.52%, and AUC of 0.420 at a cutoff point of 3,486.50 pg/mL. PlGF had a higher sensitivity of 81.25%; however, it showed low specificity of 51.97% and low PPV of 17.57%. The sFlt-1/PlGF ratio had a sensitivity of 87.50%, specificity of 51.97%, PPV of 18.67%, NPV of 97.06%, DA of 55.9%, and AUC of 0.678 at a cutoff level of 8.11. The diagnostic performance of sFlt-1, PlGF, and the sFlt-1/PlGF ratio is presented in Table 2.

**Table 2:** Diagnostic performance of angiogenic biomarkers. sFlt-1, soluble fms-like tyrosine kinase-1; PlGF, placental growth factor; NPV, negative predictive value; PPV, positive predictive value; SE, standard error

Diagnostic parameter	sFlt-1 for Group A	PlGF for Group A	sFlt-1/PlGF ratio for Group A	sFlt-1 for Group B	PlGF for Group B	sFlt-1/PlGF ratio for Group B
Cutoff value	5,212 pg/mL	87.32 pg/mL	77.65	3,486.50 pg/mL	314.55 pg/mL	8.11
Sensitivity (%)	80.70	77.19	80.70	6.25	81.25	87.50
Specificity (%)	80.31	84.25	91.34	59.84	51.97	51.97
PPV (%)	64.79	68.75	80.70	1.92	17.57	18.67
NPV (%)	90.27	89.17	91.34	83.52	95.65	97.06
Area under the curve (AUC) (SE)	0.862 (0.055)	0.852 (0.030)	0.902 (0.026)	0.420 (0.055)	0.687 (0.062)	0.678 (0.060)

ROC curve findings

**Figure 1:**
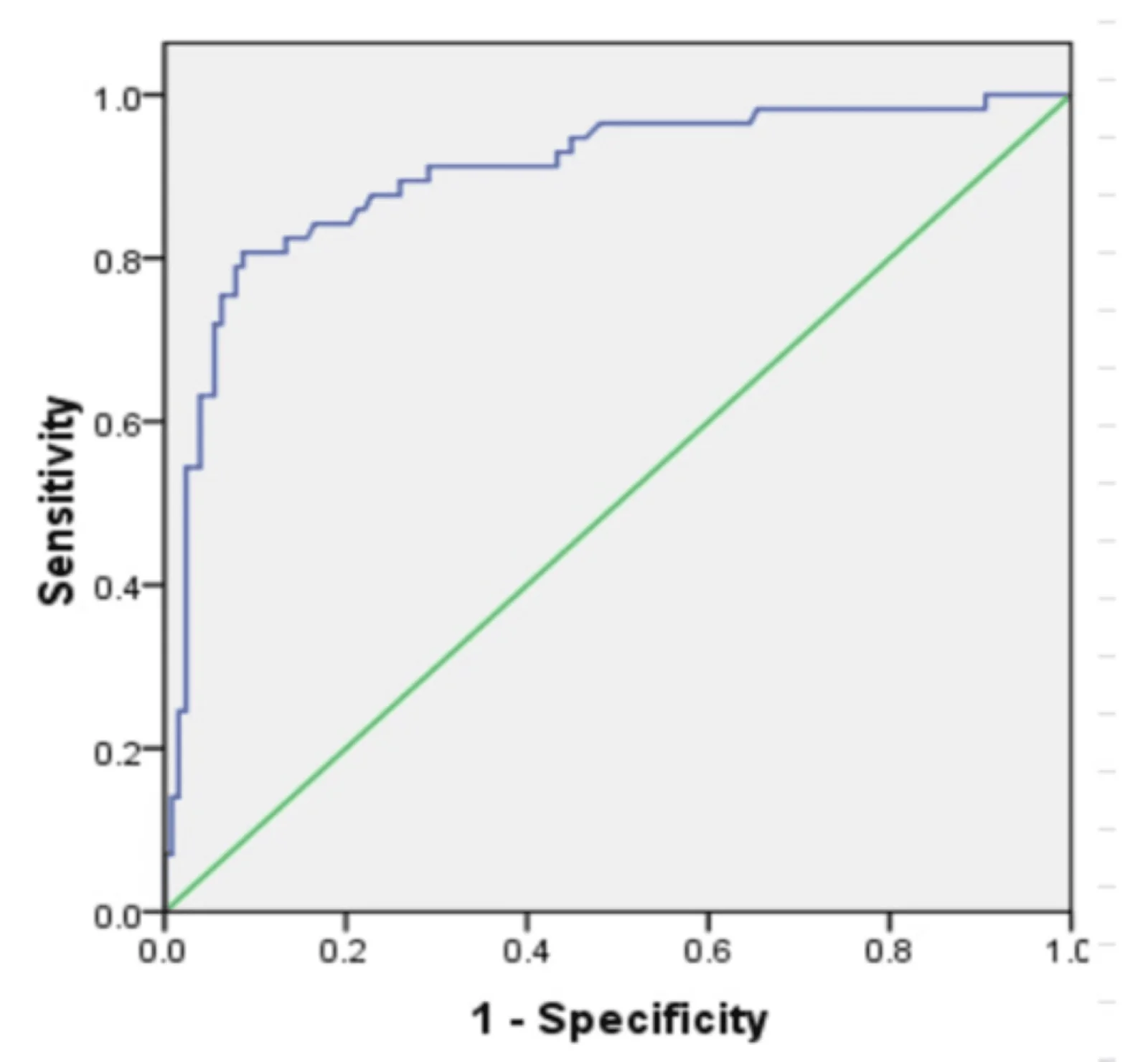
Receiver operating characteristic curve of the sFlt-1/PlGF ratio for preeclampsia. sFlt-1, soluble fms-like tyrosine kinase-1; PlGF, placental growth factor

Figure 1 shows the ROC curve of the sFlt-1/PlGF ratio for preeclampsia, demonstrating strong discriminatory performance.

**Figure 2:**
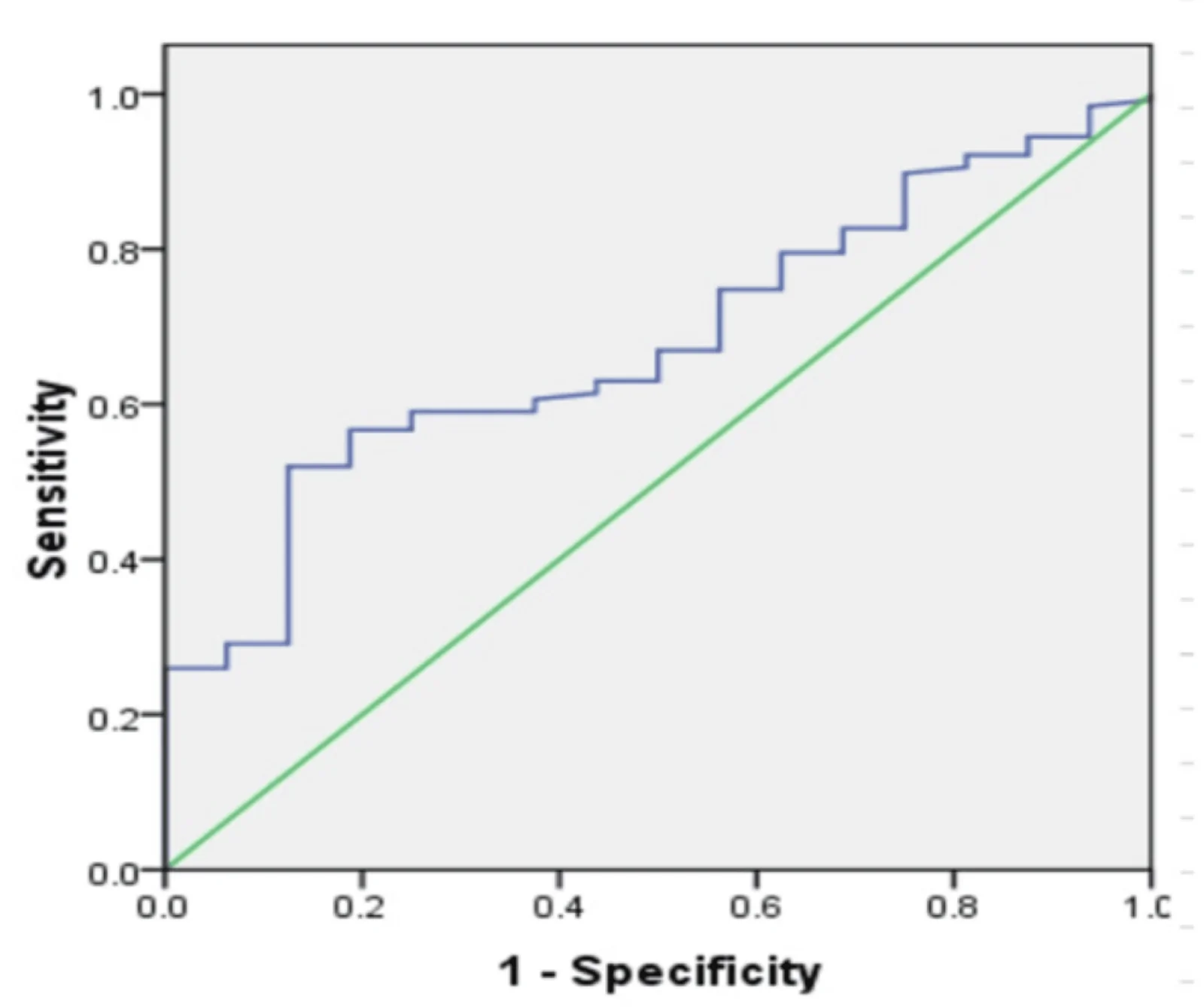
Receiver operating characteristic curve of the sFlt-1/PlGF ratio for placenta accreta spectrum. sFlt-1, soluble fms-like tyrosine kinase-1; PlGF, placental growth factor

Figure 2 shows the ROC curve of the sFlt-1/PlGF ratio for PAS, demonstrating comparatively weaker diagnostic performance.

Overall, angiogenic biomarkers performed better in predicting preeclampsia than PAS. The sFlt-1/PlGF ratio was the strongest predictor of preeclampsia, showing high specificity, high NPV, and good overall diagnostic accuracy. In contrast, its performance in PAS was limited by low specificity and low PPV, indicating that it is not useful as a stand-alone diagnostic marker for PAS.

## Discussion

To differentiate preeclampsia and placenta accreta spectrum (PAS) from pregnancies without either condition, this study compared the diagnostic performance of sFlt-1, PlGF, and the sFlt-1/PlGF ratio. The main finding was that the sFlt-1/PlGF ratio showed strong discriminatory performance for preeclampsia but limited and weak diagnostic performance for PAS. For preeclampsia, the sFlt-1/PlGF ratio showed the highest area under the curve among the evaluated biomarkers. At a cutoff value of 77.65, it demonstrated a sensitivity of 80.70%, specificity of 91.34%, PPV of 80.70%, NPV of 91.34%, diagnostic accuracy of 88.0%, and an area under the curve of 0.902. For PAS, the ratio had high sensitivity and NPV but low specificity and PPV, making it unsuitable as a stand-alone marker. Given the small PAS sample and marked group imbalance, these PAS findings should be interpreted as exploratory and hypothesis-generating rather than practice-changing.

The better performance of the sFlt-1/PlGF ratio in preeclampsia is biologically coherent with the known pathophysiology of preeclampsia. Key abnormalities in preeclampsia include abnormal trophoblastic invasion, failure of spiral artery remodeling, placental ischemia, oxidative stress, and systemic endothelial dysfunction. These processes upregulate antiangiogenic factors such as sFlt-1 and downregulate proangiogenic factors such as PlGF in circulation [14]. The sFlt-1/PlGF ratio is thus a composite marker of angiogenic imbalance, which may explain why it performed better than sFlt-1 and PlGF individually. The observed cutoff value of 77.65 was derived from the present cohort and should not be considered universally applicable without external validation, although it is broadly consistent with previously reported diagnostic and short-term predictive thresholds in women with suspected preeclampsia [15,16]. The shorter interval between biomarker testing and delivery in Group A further suggests that a high ratio may identify clinically significant disease requiring closer monitoring and timely intervention. In routine practice, the ratio should be interpreted alongside blood pressure measurements, proteinuria assessment, laboratory evaluation, fetal surveillance, and the overall maternal clinical condition rather than used in isolation.

A higher frequency of adverse fetal outcomes, including fetal growth restriction, oligohydramnios, intrauterine demise, and NICU admission, was observed in the preeclampsia group. These findings are consistent with placental insufficiency, reduced uteroplacental perfusion, fetal hypoxia, impaired fetal growth, and reduced amniotic fluid volume. Previously, such associations have been reported between preeclampsia and fetal growth restriction [12]. Increased NICU admission may reflect fetal compromise, medically indicated preterm delivery, or both. Although several fetal outcomes differed significantly among the groups, these results should be interpreted cautiously, particularly for the PAS group, because of its small sample size and the low number of outcome events. Neonatal mortality and prolonged hospitalization did not differ significantly among the groups.

The findings for PAS differed from those observed for preeclampsia. Although the sFlt-1/PlGF ratio showed high sensitivity and NPV at a cutoff value of 8.11, it had low specificity and PPV, indicating that it is unsuitable for confirming PAS as a stand-alone diagnostic test. The AUC of 0.678 indicates weak discriminatory performance and limits the clinical usefulness of the PAS-derived threshold. The poor diagnostic performance is probably related to the distinct pathophysiology of PAS, which is primarily characterized by defective decidualization, abnormal placental adherence, and varying degrees of myometrial invasion rather than the diffuse placental ischemia and systemic endothelial dysfunction characteristic of preeclampsia. Consequently, the marked angiogenic imbalance observed in preeclampsia may be absent, less pronounced, or more heterogeneous in PAS. These findings corroborate previous studies indicating that angiogenic markers are not useful as stand-alone diagnostic tools in PAS [17-19]. Established clinical risk factors, particularly placenta previa, prior cesarean delivery, previous uterine surgery, and maternal age, remain central to PAS risk assessment.

These findings have distinct clinical implications for the two conditions. In suspected preeclampsia, the sFlt-1/PlGF ratio may be used as an adjunct to clinical assessment, blood pressure monitoring, proteinuria assessment, laboratory testing, and fetal surveillance. A clinically abnormal ratio may support decisions regarding intensified surveillance, specialist assessment, repeat maternal and fetal evaluation, and timing of delivery, but it should not independently determine management. PAS diagnosis should be based mainly on clinical risk factors and imaging, particularly transabdominal and transvaginal ultrasonography with color Doppler. Magnetic resonance imaging may be helpful when ultrasound findings are inconclusive, posterior placentation is present, or deeper invasion is suspected. Angiogenic biomarkers should therefore be positioned only as potential adjuncts to imaging and clinical risk assessment in PAS, not as replacements for them.

Strengths, limitations, and future directions

The strengths of this study include its prospective design, standardized measurement of sFlt-1 and PlGF, assessment of multiple diagnostic indices, and follow-up through delivery. It also compared angiogenic biomarkers in preeclampsia and PAS, two placentation disorders with distinct pathophysiology.

The main limitations were the small PAS group (*n* = 16), marked group imbalance, and single-center design, which reduced the precision and generalizability of PAS estimates. PAS findings should therefore be considered exploratory and hypothesis-generating. Biomarkers were measured at a single time point, preventing assessment of longitudinal changes. No multivariable adjustment was performed for maternal age, gestational age at testing, socioeconomic status, disease severity, placenta previa, prior cesarean delivery, or other PAS risk factors. The cohort-derived cutoffs were not externally validated, and fetal outcomes involving the PAS group should be interpreted cautiously.

Larger multicenter studies with balanced group sizes, serial biomarker measurements, multivariable modelling, and external validation are required. Future studies should evaluate whether angiogenic biomarkers provide incremental diagnostic value when combined with established clinical risk factors and imaging rather than assessing them as stand-alone tests for PAS.

## Conclusions

This study shows that angiogenic biomarkers, particularly the sFlt-1/PlGF ratio, have strong discriminatory value for preeclampsia but limited diagnostic value for PAS. Among the evaluated markers, the sFlt-1/PlGF ratio showed the highest sensitivity, specificity, PPV, NPV, and diagnostic accuracy for preeclampsia. These findings support its use as an adjunct to blood pressure assessment, proteinuria testing, laboratory evaluation, and fetal surveillance rather than as an isolated diagnostic test. Fetal growth restriction, oligohydramnios, intrauterine demise, and NICU admission were more frequent among women with preeclampsia, emphasizing the importance of early detection and close monitoring. For PAS, the sFlt-1/PlGF ratio showed high sensitivity and NPV but low specificity, low PPV, and weak overall discriminatory performance. Given the small PAS sample, group imbalance, absence of multivariable adjustment, and cohort-derived cutoff values, these findings should be considered exploratory and hypothesis-generating rather than practice-changing. PAS diagnosis should continue to rely primarily on established clinical risk factors and imaging. Larger multicenter studies with balanced groups, serial biomarker measurements, confounder adjustment, and external validation are required before these cutoffs can be applied clinically.

## References

[1] Macedo TC, Montagna E, Trevisan CM (2020). Prevalence of preeclampsia and eclampsia in adolescent pregnancy: a systematic review and meta-analysis of 291,247 adolescents worldwide since 1969. Eur J Obstet Gynecol Reprod Biol.

[2] Belay AS, Wudad T (2019). Prevalence and associated factors of pre-eclampsia among pregnant women attending anti-natal care at Mettu Karl referal hospital, Ethiopia: cross-sectional study. Clin Hypertens.

[3] Ndwiga C, Odwe G, Pooja S, Ogutu O, Osoti A, E Warren C (2020). Clinical presentation and outcomes of pre-eclampsia and eclampsia at a national hospital, Kenya: a retrospective cohort study. PLoS One.

[4] Magee LA, Nicolaides KH, von Dadelszen P (2022). Preeclampsia. N Engl J Med.

[5] Pegu B, Thiagaraju C, Nayak D, Subbaiah M (2021). Placenta accreta spectrum-a catastrophic situation in obstetrics. Obstet Gynecol Sci.

[6] Dar P, Doulaveris G (2024). First-trimester screening for placenta accreta spectrum. Am J Obstet Gynecol MFM.

[7] Collins SL, Ashcroft A, Braun T (2016). Proposal for standardized ultrasound descriptors of abnormally invasive placenta (AIP). Ultrasound Obstet Gynecol.

[8] Belfort MA (2010). Placenta accreta. Am J Obstet Gynecol.

[9] Birdir C, Droste L, Fox L (2018). Predictive value of sFlt-1, PlGF, sFlt-1/PlGF ratio and PAPP-A for late-onset preeclampsia and IUGR between 32 and 37 weeks of pregnancy. Pregnancy Hypertens.

[10] Zhao H, Wong RJ, Stevenson DK (2021). The impact of hypoxia in early pregnancy on placental cells. Int J Mol Sci.

[11] Kapoor H, Hanaoka M, Dawkins A, Khurana A (2021). Review of MRI imaging for placenta accreta spectrum: pathophysiologic insights, imaging signs, and recent developments. Placenta.

[12] Wisner K (2019). Gestational hypertension and preeclampsia. MCN Am J Matern Child Nurs.

[13] Taneja A, Kaur G, Bansal E, Handa S, Gupta S (2025). Predictive value of the sFlt-1/PlGF ratio in pre-eclampsia and placenta accreta spectrum: a prospective observational study [PREPRINT]. Authorea Preprints.

[14] Xia Y, Wang Y, Yuan S (2024). Development and validation of nomograms to predict clinical outcomes of preeclampsia. Front Endocrinol (Lausanne).

[15] Hesson A, Langen E (2018). Outcomes in oligohydramnios: the role of etiology in predicting pulmonary morbidity/mortality. J Perinat Med.

[16] Pan X, Peng J, Chen Y, Di X, Li P, Zhang G, Liu H (2025). sFlt-1/PlGF ratio thresholds for diagnosing pre-eclampsia in pregnant women with high blood pressure. Ultrasound Obstet Gynecol.

[17] Wihakarat A, Singkhamanan K, Pranpanus S (2024). Antenatal maternal serum biomarkers as a predictor for placenta accreta spectrum disorders. Placenta.

[18] Zhu X, Chen L, Li R (2020). Values of serum sFlt-1, PLGF levels, and sFlt-1/PLGF ratio in diagnosis and prognosis evaluation of preeclamptic patients. Clin Exp Hypertens.

[19] Verlohren S, Brennecke SP, Galindo A (2022). Clinical interpretation and implementation of the sFlt-1/PlGF ratio in the prediction, diagnosis and management of preeclampsia. Pregnancy Hypertens.

